# Prevention of Endotoxaemia in Obstructive Jaundice —  a Comparative Study of Bile Salts

**DOI:** 10.1155/1988/42486

**Published:** 1988

**Authors:** J. A. Pain, M. E. Bailey

**Affiliations:** 1 Department of Surgery Royal Surrey County Hospital Surrey Guildford UK; 2 FRCS Department of Surgery Mayday Hosptial Thornton Heath, Croydon CR4 7YE UK

## Abstract

Systemic endotoxaemia is associated with postoperative renal dysfunction in obstructive jaundice, and can be prevented by the pre-operative administration of certain bile salts. In order to find the most effective bile salt for use in this condition, a comparison of the anti-endotoxic activities of different bile salts was performed. Bile salts were incubated *in vitro* with endotoxin and the resultant endotoxin level was measured with a quantitative limulus assay. The *in vivo* effects of different oral bile salts on the intestinal absorption of radiolabelled endotoxin from rats with obstructive jaundice were compared. The *in vitro* and *in vivo* anti-endotoxic activities of bile salts related to their known detergent activities. Deoxycholic acid and its conjugates were the most effective and should be the bile salts of choice for further clinical evaluation in obstructive jaundice in man.

### INTRODUCTION

Postoperative acute renal failure occurs in 9% of patients with obstructive jaundice[1]. There is considerable evidence to implicate the presence of endotoxins within the systemic circulation in the development of this renal dysfunction[2–4]. Systemic endotoxaemia is believed to result from an increased absorption of intestinal endotoxin into the portal circulation[4,5] and its impaired removal by the Kupffer cells within the liver[6,7].

The presence of bile within the intestinal lumen reduces endotoxin absorption[8,9]. Oral administration of bile salts abolishes endotoxaemia in rats with bile-duct ligation[4]. In patients with obstructive jaundice, oral sodium deoxycholate prevents both portal and systemic endotoxaemia and protects postoperative renal function[10]. However, chenodeoxycholic acid is less effective in these roles[11] and ursodeoxycholic acid has been found to confer no benefit[12]. An *in vitro* and *in vivo* comparison of bile salts has been performed to find which bile salt should be the most effective anti-endotoxaemic agent for use in obstructive jaundice.

## METHODS

### *In Vitro Study*

Different bile salts and their conjugates were compared as to their ability to inhibit endotoxin activity *in vitro*. Bile salts were incubated with endotoxin and the resultant endotoxin level was measured.

The bile salts (200 mg, >98% purity, Sigma) were dissolved in 99% ethanol (4 ml), and then added to sodium phosphate buffer 86 ml, pH 7.0). Various concentration of the bile-salt/buffer solution were mixed with *Escherichia coli* endotoxin (20 μg, 0111B4, Difco) in water (0.2 ml), made up to 2 ml with sodium phosphate buffer and incubated at 37°C for 6 h. The mixtures were then assayed for endotoxin by means of a quantitative chromogenic limulus assay[5]. The assay had a lower limit of sensitivity of 1pg/ml endotoxin in water, with a coefficient of variation of 4% with 50pg/ml endotoxin in water. All tests were performed in triplicate with appropriate controls.

### *In Vivo Study*

Different bile salts and their conjugates were compared in their ability to prevent the absorption of radiolabelled endotoxin from the intestines of rats with obstructive jaundice.

Endotoxin was labelled with $Cr^{51}$ according to the method of Braude *et al.*[13] Endotoxin, E.coli 0111B4 (700 mg, Sigma) was suspended in phosphate chloride buffer (140 ml, pH 7). The endotoxin suspension was incubated at 37°C for 24 h with radiolabelled sodium chromate (300 MBq, Amersham International). After incubation the endotoxin/sodium chromate was dialysed against de-ionized water, weakly buffered with phosphate chloride, at room temperature, until there was no longer any significant loss of radioactivity into the dialysate. The radiolabelled endotoxin was stored at 4°C in gas-evacuated vials and, prior to use, tested for unbound chromium by further dialysis.

Seventy-four male Wistar rats (weight 270–300gm) underwent common bile-duct (CBD) ligation and division or sham operation on Day 0 and were treated as shown in Figure 1. The extrapancreatic part of the CBD was mobilized and divided between silk ligatures, and in the sham operation a silk ligature was placed around the CBD and then removed.

On Day 12, 63 rats (seven groups of nine) received one of the following bile salts in an oral dose of 6mg/100gm body weight every 8 h for two days: sodium deoxycholate, cholic acid, chenodeoxycholic acid, ursodeoxycholic acid, sodium taurocholate, sodium taurodeoxycholate, and glycodeoxycholic acid. A dose of 6mg bile salt/100 gm body weight was chosen since preliminary experiments had shown that this dose of sodium deoxycholate had prevented the absorption of more than 60% of orally administered radiolabelled endotoxin. On Day 14, 4 h after the final dose of bile salts, radiolabelled endotoxin (2mg/100gm body weight) was given via a gavage tube to all rats.

Four hours later the rats were killed by cervical dislocation, and immediate laparotomy was performed. Ligatures were placed around the lower oesophagus and distal rectum, and the intestines including the stomach were removed. The attached mesentery was discarded and the intestines were counted for radioactivity to calculate the percentage radioactivity remaining in the intestines. From three control jaundiced rats the livers, lungs, spleens, kidneys, and samples of blood and urine were also measured for radioactivity.

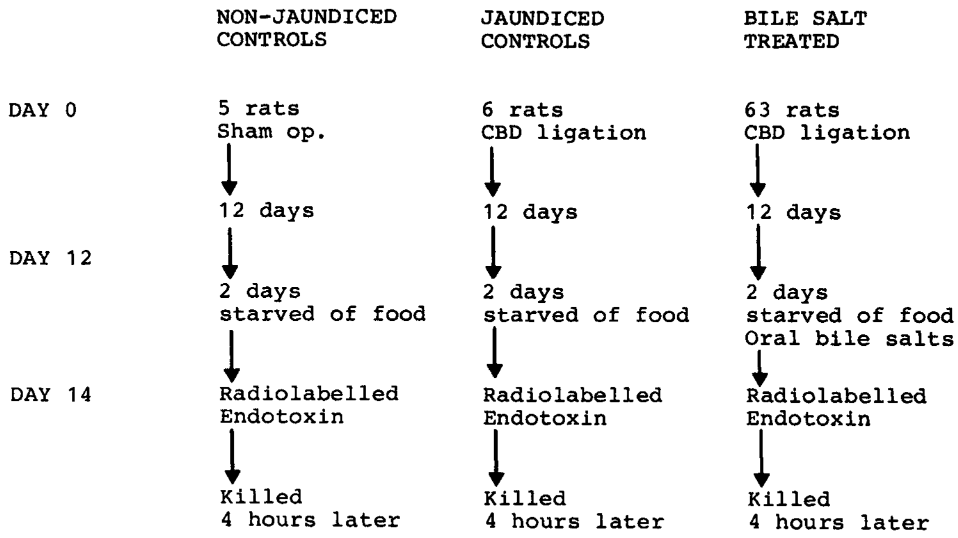

**Figure 1** Design of animal experiment.

#### *Statistics*

Comparisons between groups were made with.the Student's t-test.

### RESULTS

#### *In Vitro Study*

Controls in which only buffer was added to endotoxin showed no significant loss of detectable endotoxin after incubation. All bile salts tested reduced the detectable endotoxin level. The dihydroxy bile salts, glycodeoxycholic acid and deoxycholic acid, proved to have the greatest *in vitro* anti-endotoxic action. Only 400μg/ml of these bile salts was required to reduce the detectable endotoxin level from 10μg/ml to <50pg/ml (20 000-fold decrease). The mean activities of other bile salts expressed as a percentage of that of deoxycholic acid are shown in Table 1.

#### *In Vivo Study*

The rats each received 1.7ml radiolabelled endotoxin, which produced a mean 149 781 counts per minute. No diarrheogenic effect of the bile salts was evident. Two rats in the chenodeoxycholic-acid-treated group and one in the taurocholate-treated group died during the experiment and are not included in the results. In the sham-operated (non-jaundiced) rats less than 10% (mean intestinal radioactivity count 137 079 ±7373) of the radioactive label was absorbed, whereas in the jaundiced rats 65% (mean intestinal radioactivity count 52 425 ±7193) was absorbed (t = 19.2, d.f. = 9, $P<0.001$). The mean radioactivity counts per minute of the tissue and body fluids taken from the three jaundiced control rats were liver 13 259, spleen 982, kidney 4873, urine 2240, packed cell blood 1871 per ml, plasma 724 per ml.

All bile salts tested, except ursodeoxyxholic acid, produced a significant ($P<0.05$)

**Table 1** *In vitro* and *in vivo* comparison of bile salts. *In vitro* results expressed as bile-salt concentration required to reduce the detectable endotoxin level from 10μg/ml to 50pg/ml. *In vivo* results expressed as mean ±s.d. residual intestinal endotoxin radioactivity counts. In bracket: percentage activity compared to glycodeoxycholic acid.

| | *In Vitro* | | *In Vivo* | | |
|---|---|---|---|---|---|
| | *μg/ml* | % | $\times 10^3$ | *cpm* | % |
| Secondary bile salts | | | | | |
| Glycodeoxycholic acid | 400 | (100) | 114 | ±12 | (100) |
| Deoxycholic acid | 400 | (100) | 116 | ±18 | (97) |
| Na Glycodeoxycholate | 500 | (80) | | | |
| Na Taurodeoxycholate | 500 | (80) | 106 | ±23 | (90) |
| Chenodeoxycholic acid | 600 | (66) | 105 | ±16 | (89) |
| Na Glycochenodeoxycholate | 600 | (66) | | | |
| Na Taurochenodeoxycholate | 700 | (57) | | | |
| Primary bile salts | | | | | |
| Cholic acid | 1700 | (23) | 89 | ±23[a] | (79) |
| Glychocolic acid | 1700 | (23) | | | |
| Na Glycocholate | <2000 | (>20) | | | |
| Na Taurocholate | <2000 | (>20) | 84 | ±19[b] | (74) |
| Tertiary bile salts | | | | | |
| Ursodeoxycholic acid | <2000 | (>20) | 70 | ±23[c] | (62) |

Student's t-test [a]$P>0.05$
[b]$P>0.01$
[c]$P>0.001$

reduction in the absorption of the radiolabelled endotoxin. Glycodeoxycholic acid was the most effective agent and reduced the percentage endotoxin absorption to a mean 24 ±8. The activities of other bile salts expressed as a percentage of that of glycodeoxycholic acid are shown in Table 1.

### DISCUSSION

Bile salts are detergents and have a direct anti-endotoxic action *in vitro*, causing inactivation of the endotoxin molecule[14–16]. Endotoxin, which is aggregated into particles with a molecular weight of about 1 000 000, is disassociated by bile salts to form non-toxic subunits with molecular weights of about 20 000[14].

The presence of bile within the intestinal lumen prevents endotoxin absorption. The clinical effects of endotoxaemia cannot be induced in healthy animals by the oral administration of endotoxin even if doses 500–3000 times the minimal lethal parenteral dose are given[17,18]. Endotoxin absorption is increased in obstructive jaundice[4] or after bile-duct cannulation and external biliary drainage[8], but is reduced in obstructive jaundice if the endotoxin is incubated with a bile salt prior to its oral administration[19], if oral bile salts are administered[4], or if internal biliary drainage is performed[9].

All bile salts tested had potent *in vitro* anti-endotoxic activities, and the most active were those of the dihydroxy bile acid, deoxycholic acid and its conjugates, and the least activity was shown by ursodeoxycholic acid. Lithocholic acid and its conjugates were not tested since they are toxic and would be unsuitable for use in man. These results are consistent with previous studies examining the *in vitro* anti-endotoxic activity of bile salts. Iwasaki and Tanikawa[20] found that deoxycholic acid was the most effective bile salt compared to chenodeoxycholic acid, its conjugates and taurocholic acid. Liehr *et al.* [21], comparing conjugates of bile salts, showed that those of deoxycholate had a greater anti-endotoxic activity than those of chenodeoxycholate, while those of cholate were the least effective. These previous studies measured endotoxic with a semiquantitative limulus assay based on a subjective assessment of the type of gel formed. The assay used in the present experiment was highly sensitive, reproducible and quantitative.

The comparative *in vitro* bile-salt activities related to their known critical micellar concentration[22,23], which supports the theory that it is the detergent properties of the bile salts that render endotoxins inactive.

The labelling of endotoxin with $Cr^{51}$ was first described by Braude *et al.*[13] Although there is uncertainty as to the exact location of the radiolabel within the endotoxin molecule, the attachment is very stable both *in vitro* and *in vivo*[13,24–26]. Four hours after oral administration of the radiolabelled endotoxin to the jaundiced rats, some absorbed radioactivity was in the liver, but most was present in the plasma. This distribution is similar to that found after intravenous injection of $Cr^{51}$ -labelled endotoxin into animals[13]. Endotoxin binds to plasma high-density lipoproteins and circulates in this complex form until cleared from the ciruclation[27]. In our study it is unlikely that the $Cr^{51}$ present in the circulation was free, because if free $Cr^{51}$ is injected intravenously the majority can subsequently be found within the red-cell layer and not within the plasma[28,29].

There was increased absorption of radiolabelled endotoxin in the bile-duct-ligated rat. This probably occurred due to a lack of intestinal bile rather than due to the hyperbilirubinaemic state, since bile-duct cannulation in rats also leads to an increased absorption of endotoxin[8,9]. All of the bile salts tested, except for ursodeoxycholic acid, significantly reduced endotoxin absorption. As in the *in vitro* study deoxycholic acid and its conjugates were the most effective, and the bile-salt activities related to their known detergent activities[20,21]. A greater difference between the bile-salt activities was shown by the *in vitro* test, but this probably related to the bile salts being incubated with the endotoxin for 6 h in controlled conditions, whereas in the *in vivo* test the endotoxin was given only 4 h before the animal was killed; also, numerous biological variables could effect the interaction of bile salts and endotoxin. The choice of the most suitable bile salt as a prophylactic agent against endotoxaemia in obstructive jaundice depends not only on the individual salt's activity as determined *in vitro* but also on its metabolism and action *in vivo*. It is probably unimportant whether a bile "acid" or bile "salt" is used, since most bile "salts" precipitate as bile "acids" in the stomach as their precipitation pH values are higher than the luminal pH[30].

In order to prevent endotoxaemia in obstructive jaundice it is recommended that deoxycholic acid or its conjugates be the agents of choice for further clinical evaluation.
